# Study of the Chemical Profile and Anti-Fungal Activity against *Candida auris* of *Cinnamomum cassia* Essential Oil and of Its Nano-Formulations Based on Polycaprolactone

**DOI:** 10.3390/plants12020358

**Published:** 2023-01-12

**Authors:** Roberto Rosato, Edoardo Napoli, Giuseppe Granata, Maura Di Vito, Stefania Garzoli, Corrada Geraci, Silvia Rizzo, Riccardo Torelli, Maurizio Sanguinetti, Francesca Bugli

**Affiliations:** 1 Dipartimento di Scienze Biotecnologiche di Base, Cliniche Intensivologiche e Perioperatorie, Università Cattolica del Sacro Cuore, 00167 Rome, Italy; 2 Istituto di Chimica Biomolecolare—Consiglio Nazionale delle Ricerche, 95126 Catania, Italy; 3 Dipartimento di Chimica e Tecnologie del Farmaco, Università di Roma Sapienza, Piazzale Aldo Moro 5, 00100 Rome, Italy; 4 Dipartimento di Scienze di Laboratorio e Infettivologiche, Fondazione Policlinico Universitario A. Gemelli IRCCS, Largo A. Gemelli 8, 00168 Rome, Italy

**Keywords:** cinnamaldehyde, cinnamon, antifungal properties

## Abstract

Background: *Candida auris* represents an emerging pathogen that results in nosocomial infections and is considered a serious global health problem. The aim of this work was to evaluate the in vitro antifungal efficacy of *Cinnamomum cassia* essential oil (CC-EO) pure or formulated in polycaprolactone (PCL) nanoparticles against ten clinical strains of *C. auris*. Methods: nanoparticles of PCL were produced using CC-EO (nano-CC-EO) and cinnamaldehyde (CIN) through the nanoprecipitation method. The chemical profile of both CC-EO and nano-CC-EO was evaluated using SPME sampling followed by GC-MS analysis. Micro-broth dilution tests were performed to evaluate both fungistatic and fungicidal effectiveness of CC-EO and CIN, pure and nano-formulated. Furthermore, checkerboard tests to evaluate the synergistic action of CC-EO or nano-CC-EO with micafungin or fluconazole were conducted. Finally, the biofilm disrupting activity of both formulations was evaluated. Results: GC-MS analysis shows a different composition between CC-EO and nano-CC-EO. Moreover, the microbiological analyses do not show any variation in antifungal effectiveness either towards the planktonic form (MIC_CC-EO_ = 0.01 ± 0.01 and MIC_nano-CC-EO_ = 0.02 ± 0.01) or the biofilm form. No synergistic activity with the antifungal drugs tested was found. Conclusions: both CC-EO and nano-CC-EO show the same antimicrobial effectiveness and are potential assets in the fight against *C. auris.*

## 1. Introduction

First isolated in 2009, *Candida auris* has rapidly become a multidrug-resistant fungus of interest to global public health [1]. Several factors have made this fungus particularly harmful to human health, including the diagnostic difficulty with common laboratory methods, the resistance to current anti-fungals, the presence of virulence factors, and the ability to colonize surfaces and medical devices. The above has recently allowed the spread of nosocomial infections and outbreaks with high mortality rates [2].

In May 2018, the Italian Ministry of Health promptly issued the ECDC’s Rapid Risk Assessment and then subsequent recommendations aimed at raising awareness of this pathogen to increase surveillance and improve case management were issued [3,4,5]. In July 2022, given the spread of *C. auris* to various healthcare facilities in Liguria and interregional spread to Emilia-Romagna, in order to contain any outbreaks and to deal with any national and international alert, the Ministry of Health identified the Complex Operating Unit of Microbiology of Policlinico Gemelli IRCCS in Rome (the laboratory of the microbiologists authors of this study) as a National Reference Laboratory for the characterization of *C. auris* isolates and the collection of clinical strains [6].

Resistance to antifungals, normally used as drugs or for surface treatment, is the basis of the high virulence and mortality. Most of the isolated strains are resistant to at least one class of antifungals, such as azoles, commonly used in therapy, and many of these isolates show multiple resistance, sometimes to all available drugs [6].

This microorganism essentially affects immunocompromised people in nosocomial healthcare [7], and recently several infections have been found among newborns and/or premature infants [8].

Therefore, appropriate control/containment measures using suitable room disinfection protocols are essential to reduce the spread. It has been demonstrated that chlorine-based products are the most effective for the disinfection of environments, while few and conflicting data are published regarding the efficacy of antiseptics (e.g., chlorhexidine) for decolonization of patients and hygiene of the hands of the healthcare personnel [9]. Therefore, the control of *C. auris* is a challenge where laboratories, physicians, public health agencies and scientific research are needed; the former to identify and treat infections and prevent transmission, the latter to identify valid alternatives to current treatments capable of overcoming the resistance of the fungus.

There are many lines of research aimed at identifying new resources in the fight against antibiotic resistance of *C. auris*. Drug reuse, combinations of new or old drugs, vaccine development, research of new synthetic (small molecules, new echinocandins, antimicrobial peptides, monoclonal antibodies) or natural (small molecules, peptides, plant extracts) compounds [10,11] are some of the many lines of research for the discovery and development of new drugs.

The essential oils (EOs) produced by the distillation of aromatic plants are, among the natural substances, the products with the highest antimicrobial potential [12,13]. Several studies have been recently published to evaluate the antifungal activity of EOs against *C. auris* and its biofilm [14,15,16,17], and the *Cinamomum cassia* EO (CC-EO) is one of the most powerful with its bioactive compound cinnamaldehyde [18,19]. *C. cassia* Presl is a tropical aromatic evergreen tree of the Lauraceae family, commonly used in traditional Chinese medicine. It is also a traditional spice, widely used around the world. From its bark, it is possible to obtain an EO characterized by 70–90% cinnamaldehyde which defines its interesting anti-bacterial and anti-fungal activities [20].

Recent findings again prompt that EOs can function in a synergistic combination with antifungals [21,22]. Regarding the delivery of EOs, in the last decade, polymeric nanoparticles have been extensively investigated as a controlled release systems for different hydrophobic substances [23,24,25,26,27]. Moreover, nano-encapsulation of bioactive compounds of EOs introduces several advantages such as the protection from the external environment, the improvement of the stability and bioavailability. Few authors evaluated the effectiveness of some EOs when carried in liposomes or nanocapsules [23,24].

Among the different polymeric materials, polycaprolactone (PCL) possesses excellent physicochemical characteristics for controlled release due to its high permeability to a wide range of drugs, a peculiar non-toxicity and a low degradation rate. Its good solubility in many solvents and its exceptional compatibility with various mixtures, including EOs [28,29], has allowed it to be widely used in various medical applications [30,31]. These advantages have identified interesting uses in the preparation of polymeric microparticles and nanoparticles, scaffolds and tissues [32,33,34].

The aim of this article is to evaluate and compare the antifungal efficacy of *C. cassia* EO and its nano formulation in PCL particles against clinical strains of *C. auris* by evaluating fungistatic and fungicidal activity.

## 2. Results

### 2.1. Preparation and Physicochemical Characterization of Nano-CC-EO and Nano-CIN

The properties of nanoparticles, such as better selectivity and the ability to cross tissue barriers, are leading to the development of new techniques in the pharmacological field, for the development of innovative therapies. In the biomedical sector, one of the main applications concerns the formulation of new systems for the administration of drugs through nanoparticles in drug delivery systems. In this regard, monodisperse nanoparticles find interesting applications in nanomedicine [35]. The interfacial deposition of the preformed polymer method [36] is an effective strategy to nanoencapsulate essential oils [28,29,37]. This method with small variations [28] was used to prepare the nanocapsules loaded with CC-EO or CIN reported in this work. It consists in the addition of a surfactant aqueous solution to an organic phase, formed by polymer, dispersant and bioactive (CC-EO or CIN) dissolved in acetone. For diffusion of the organic solvent into the aqueous phase and subsequent its removal by vacuum evaporation, a lactescent colloidal solution containing nanocapsules is formed. The choice of a biodegradable and biocompatible polymer such as poly(ε-caprolactone) together with the use of a non-ionic and non-toxic surfactant such as polysorbate 80, that prevents unwanted coalescence and precipitation phenomena, it is essential for obtaining of green, stable, and robust nanosystems. Moreover, this method is easy-to-perform, highly reproducible, and cheap due to the low cost of the materials used. In Table 1 the physicochemical values (z-average diameter, polydispersity index (PDI), zeta potential (ζ), encapsulation efficiency (EE) and loading capacity (LC)) of the nano-CC-EO and nano-CIN are shown, respectively. The nanometric diameters and the very low PDI values for both nanosuspensions were indicative of the presence in aqueous medium of substantially monodisperse nanosystems. The negative zeta potential values, due to the free polymer carboxylic residues, agreed with other stable PCL nanocapsules previously reported [28,29,37]. For the nano-CC-EO an EE of 81% was indicative of a highly efficient encapsulation process. A tendency towards lower value of EE (73%) was determined instead for nano-CIN. This can be attributed to the non-negligible solubility in water of CIN compared to the lipophilicity of the CC-EO phytocomplex. LC values around 50% for both nanosuspension confirm the excellent percentages by weight of the bioactives compared to the other components forming the nanocapsules.

### 2.2. GC-MS Analyses of EO

The chemical composition of CC-EO obtained by direct injection was characterized by 29 components listed in Table 2. *Trans*-cinnamaldehyde was the main compound of the phytocomplex (85.5%) followed by *O*-methoxycinnamaldehyde (8.6%) and other minor molecules whose percentage values ranged from 0.1% to 0.9%.

### 2.3. HS-GC-MS Analyses

Headspace sampling followed by GC-MS analysis was carried out to evaluate the release of the volatile components from the nano-CC-EO in comparison with the free CC-EO one. The obtained results listed in Table 3 show that (E)-cinnamaldehyde was the major component found in both samples. Specifically, it was 80.7% in the free CC-EO and 80.1% in the nano-CC-EO. The other compounds revealed in the nano-CC-EO with the exception of 1,8-cineole (11.0%) were detected with lower percentage values respect to free CC-EO (2.5%). In general, a higher number of components equal to 27 was detected in the free CC-EO rather than in the formulated where 17 were found. From a quantitative point of view, 1,8-cineole was present in the formulation with a percentage value of 11.0% compared to that found in free EO (2.5%). Other components followed a different trend between the two samples even though the revealed differences were minor.

### 2.4. Broth Microdilution Susceptibility Test

Table 4 shows the anti-fungal efficacy of pure CC-EO, its major component, and the two nano-encapsulated products. As shown, the average MIC and MFC values of free or nano-formulated CC-EO and CIN are almost superimposable. Specifically, the average MIC values are lower in the nano-formulated than in the pure compound (0.01 ± 0.01 and 0.04 ± 0.01, respectively). Tests conducted with empty nano-formulation confirm their antifungal ineffectiveness.

### 2.5. Checkerboard Test

Table 5 shows the data obtained by testing free or nano encapsulated CC-EO together with fluconazole or micafungin. As shown, all tested strains were found to be resistant to fluconazole, while the mean values obtained from strains resistant or sensitive to micafungin were distinguished. For drug-resistant strains it was not possible to identify the FICI values but only the FIC value relating to the free or encapsulated CC-EO. Our data show that only in presence of fluconazole the encapsulation improves the effectiveness of CC-OE (MIC_CC-EO_ = 1 ± 0.00 and MIC_nano-CC-EO_ = 0.43 ± 0.12) and MIC values of fluconazole were detectable (MIC_Fluconazole_ = 19.75 ± 20.50) even if they remained in the non-therapeutic range. In no case, a synergistic behavior was found.

### 2.6. Biofilm Eradication

**Figure 1:**
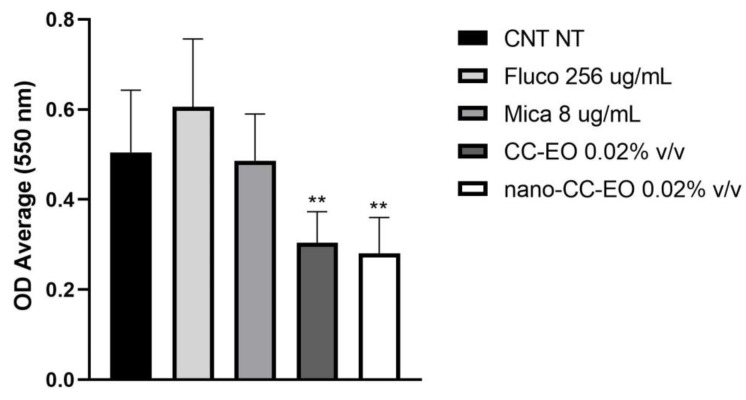
Eradication of performed biofilm. (**) indicate a statistical significance corresponding to *p* < 0.001.

Figure 1 shows the disintegrating effect of both free and nano-encapsulated CC-EO against the preformed *C. auris* biofilm. Tested conditions were characterized by Fluconazole and Micafungin at a concentration of 256 μg/mL and 8 μg/mL, respectively, and by the free and nano formulated CC-EO at the MIC values (0.02% *v*/*v*) identified by micro broth-dilution tests. All treatments made with CC-EO showed a statistically significant activity (*p* < 0.001) when compared to untreated. No difference was observed comparing untreated control (CNT NT) with samples treated with the antifungals.

## 3. Discussion

Recently, several studies have been conducted in order to identify EOs potentially active against *C. auris*. Towards *C. auris* strains, several EOs have been shown to have antifungal effectiveness when used alone (*Cymbopogon citratus*, *Eugenia caryophyllata* and *Cinnamomum zeylanicum* from bark) [16,38] or in nano formulations (*Lavandula angustifolia,* and *Lippia sidoides*) [23,24].

To our knowledge, no studies have yet evaluated the activity of CC-EO against *C. auris*. As is known, CC-EO is a cheaper species than *C. zeylanicum*, which was selected in China to counter the East India Company English monopoly that made this spice very expensive in the 17th century [39]. EOs extracted from these two species have very similar chemical profiles and, for this reason, the CC-EO is frequently used to sophisticate the EO extracted from *C. zeylanicum* EO. In 2021, Xingdong Wu et al. identified some secondary metabolites (proanthocyanidins and alkaloids) as the best markers to discriminate between the two cinnamon spices, which are otherwise difficult to distinguish even for most experts [40]. Both EOs have a strong antimicrobial activity and are characterized by cinnamaldehyde as the major component with the difference that in CC-EO it is present in a higher percentage than in the other-one. Furthermore, the lower cost of CC-EO makes it more available. For this reason, it was decided to study the antifungal activity of *C. cassia* EO alone or encapsulated in PCL-based nanocapsules in order to identify if and in which form this EO can be used in the fight against *C. auris*. As shown in Table 2, the CC-EO used in this study was characterized by a content of cinnamaldehyde and *O*-methoxycinnamaldehyde equal to 85.5% and 8.6%, respectively. These data are in line with ISO standards ISO 3216:1997 in which are specified the following standard ranges: 78.8–89.0% for cinnamaldehyde and 0.82–10.3% for *O*-methoxycinnamaldehyde [41]. PCL polymer was chosen to develop the nano-encapsulation process being a biodegradable and biocompatible polymer, useful to encapsulate a wide range of drugs. It is a promising material for the preparation of carriers with potential applications in therapeutics [31]. PCL has proved particularly suitable for the encapsulation of essential oils, improving their water solubility, their stability [37] and their biological activities [29,42]. As shown in Table 2 the nano-CC-EO has a different chemical profile compared to the free CC-EO. Analysis of both products was performed using SPME sampling followed with GC-MS analysis. Through this investigation it is possible to investigate the volatile components that are released from samples in the headspace. The results show that some chemical compounds present in the free CC-EO, in nano-CC-EO are not detected others are present with higher value percentage (e.g., 1,8-cineole present at a percentage of 11.0%compared to 2.5% in the free CC-EO), and others show a significantly lower relative amount (*O*-methoxycinnamaldehyde with a percentage of 0.7 compared to 1.0% in free CC-EO). Instead, the main chemical compound does not show significant variations (80.1% and 80.7% in the encapsulated and free CC-EO, respectively). However, these differences do not affect the antifungal effectiveness of CC-EO. Table 3 shows MIC90 and MFC90 values obtained by testing both free and nano-encapsulated CC-EO and CIN. These data show that the encapsulation process does not affect the effectiveness of EO and confirm that CIN is the active ingredient responsible for the antifungal activity also against *C. auris*. Literature data indicate that CIN exerts its antifungal action through the inhibition of ATPases, the biosynthesis of the cell wall and the alteration of the structure and integrity of the membrane [19]. To reactivate antifungal drugs, to which *C. auris* has developed resistance, checkerboard tests were performed with both free and nano-formulated CC-EO. It was decided to use CC-EO as the phytocomplex is less toxic than the active compound alone as also reported by EMA monographs [39]. Due to the antifungals resistance of the strains, it was not possible to calculate the FICI value for all tests.

The only two FICI values calculated for micafungin sensible strains indicate indifference between free or nano-formulated CC-EO when tested in combination with micafungin. Whereas, FIC_CC-EO_ values indicate absence of synergies with both fluconazole and micafungin. Only the couple between CC-EO nano-formulated and micafungin showed, against resistant strains, a synergistic value of FIC_CC-EO_ that, however, is associated with a high variability (0.38) which makes the value borderline with the additivity. This behavior is different from that observed by our group testing the *C. zeylanicum* EO which, in agreement with literature data [38], shows synergistic activity with fluconazole (data not shown). The above highlights the importance of the phytocomplex in which even the minor components play a role in defining the mechanism of action of the EO. Finally, Figure 1 shows that the nano-encapsulation process does not modify the effectiveness of CC-EO towards biofilms. In fact, data show that both CC-EO alone and nano-formulated are capable of significantly (*p* < 0.001) disrupting the biofilm if compared to antifungals that, on the contrary, fail to perturb it even if used at high concentrations. Therefore, our data show, for the first time, that the encapsulation process does not alter the properties of free CC-EO by upregulating or depressing its antimicrobial activity. This makes CC-EO encapsulated in PCL nanoparticles interesting because, as it is known in the literature, the use of polymeric and lipidic nanocapsules makes the administration of hydrophobic substances safer since, by increasing the circulating life, the solubility, the stability and the pharmacokinetic properties, reduces their systemic absorption and related side effects [43,44,45]. This is also important for compounds such as cinnamaldehyde which have a close match between therapeutic dose and toxic dose [39].

## 4. Materials and Methods

### 4.1. Chemicals

Polysorbate 80 was purchased from Fisher Chemical (Fisher Scientific, Geel, Belgium); poly(*ε*-caprolactone) (Mn 45,000) and sorbitan monostearate from Sigma–Aldrich (Milan, Italy); Water Chromasolv Plus for HPLC solvent from Honeywell Riedel-de-Haën (Seelze, Germany). All chemicals and solvents were of analytic or pharmaceutical grade.

### 4.2. Clinical Strains of C. auris

Ten *C. auris* strains, isolated from positive blood cultures, were used in antimicrobial tests, cultured in Sensititre ™ YeastOne Broth (Thermo Fisher, Waltham, MA, United States), and Sabouraud Dextrose Agar (Oxoid, Wade Road, Basingstoke, Hants, UK) were used to growth strains at 37 °C for 24 h.

### 4.3. Essential oil and Active Component

*Cinnamomum cassia* essential oil (CC-EO by Pranarôm International, Avenue des Artisans, Ghislenghien, Belgium) and cinnamaldehyde (CIN by Ventos, Barcelona, Spain, batch L4412580), its main component, were used for the study.

### 4.4. Nano-Formulations

The preparation of nano-capsules of CC-EO (nano-CC-EO) and those of cinnamaldehyde (nano-CIN) were performed by using the nanoprecipitation method as reported by Granata et al. [28]. Briefly, an organic phase, obtained by stirring at 30 °C sorbitan monostearate (35 mg), PCL (100 mg), and CC-EO or CIN (335 mg) in acetone (25 mL), was injected into the aqueous phase formed by 75 mg of polysorbate 80 dissolved in 50 mL of pure water. The mixture was stirred for 10 min at 25 °C. The organic solvent was removed carefully under vacuum to obtain nano-CC-EO or nano-CIN suspensions (50 mL). The empty nanocapsules were prepared with the same procedure but without the active ingredient.

### 4.5. Physicochemical Characterization of Nano-Capsules

Dynamic light scattering (DLS) and electrophoretic light scattering (ELS) experiments were performed at 25 °C on a Zetasizer Nano ZS-90 (Malvern Instruments, Malvern, UK). The nano-CC-EO and nano-CIN suspensions were previously diluted (1:200, *v*/*v*) with pure water or with pre-filtered (0.45 µm) 10 mM NaCl aqueous solution to perform DLS and ELS experiments, respectively. Data were analyzed using Zetasizer Version 7.02 software. DLS experiments provided the mean diameter (z-average) and polydispersity index (PDI) of nano-CC-OE and nano-CIN, while ELS provided the zeta potential (ζ). The encapsulation efficiency (EE), that represents an important parameter of the encapsulation process, was calculated using the following Equation (1):

EE (%) = (bioactive encapsulated/bioactive_tot_) × 100 (1)

where the bioactive encapsulated = bioactive_tot_ − bioactive_free_ is the amount of bioactive (CC-EO or CIN) into nano-CC-EO or nano-CIN, respectively. The total contents of CC-EO or CIN in the nanosuspensions bioactive_tot_ were determined as reported by Granata et al. [28], using an 8453 UV-Visible Spectrophotometer (Agilent Technologies). Briefly, 100 µL of each suspension were diluted with 900 µL of water and then 30 μL of this mixture were added to 3 mL of acetonitrile. The absorbance of these samples was recorded at λ_max_ 284 nm. The amount of CC-EO or CIN was derived from this value compared to the related calibration curves (R^2^ ≥ 0.9994), obtained by plotting the absorbance at 284 nm of five solutions containing different concentrations of CC-EO or CIN (from 1.4 to 6.7 µg/mL). The total content of CC-EO or CIN in the nanosuspensions were 6.0 or 5.9 mg/mL, respectively. The amount of bioactive_free_ in nanosuspension was obtained by ultrafiltration/centrifugation technique using a Heraeus Pico 21 centrifuge (Thermo Fisher Scientific, Waltham, MA, USA). Initially, 500 µL of each sample were centrifuged for 15 min at 14,000× *g* and the supernatant were transferred to Nanosep^®^ Centrifugal Devices with Omega™ Membrane 30K (Pall Life Science, Milan, Italy) and centrifuged at 3500× *g* for 180 min. Finally, to determine the free quantity of CC-EO or CIN in nanosuspension, 10 µL of ultrafiltered supernatant were diluted with 3 mL of acetonitrile and subjected to UV-visible measurements as reported above. The loading capacity (LC) of bioactive, was calculated by the following Equation (2):

LC (%) = (mass of encapsulated bioactive/mass of bioactive loaded nanocapsules) × 100(2)

### 4.6. Headspace (HS) Sampling

To investigate the vapor phase of free and nano CC-EO, a Perkin-Elmer Headspace Turbomatrix 40 autosampler connected to a Clarus 500 GC–MS was used [46,47]. To collect the volatile compounds from two samples, the operative parameters such as equilibration time and temperature were optimized.

### 4.7. GC-MS Analyses

The chemical volatile fraction of free and nano CC-EO and the liquid phase of free CC-EO, performed by direct injection, was characterized by using a Clarus 500 model Perkin Elmer (Waltham, MA, USA) gas chromatograph coupled with a mass spectrometer and equipped with a FID (flame detector ionization). For the separation of compounds, a Varian Factor Four VF-1 capillary column was housed in the GC oven. Briefly, the oven GC temperature program was: isothermal at 60 °C for 5 min, then ramped to 220 °C at a rate of 6 °C min^−1^, and finally isothermal at 220 °C for 20 min. He was used as carrier gas at flow rate of 1.0 mL min^−1^ in constant flow. The mass spectra were obtained in the electron impact mode (EI), at 70 eV in scan mode in the range 35–400 m/z. Relative percentages for quantification of the components were calculated by electronic integration of the GC-FID peak areas using the normalization method without the use of an internal standard and any factor correction. The identification of the separated compounds was performed by comparing the mass spectra for each compound with that reported on the MS library search (Wiley and Nist 02). Furthermore, linear retention index (LRI) of each compound was calculated using a mixture of *n*-alkanes (C_8_-C_30_, Ultrasci) injected directly into GC injector at the same operating conditions reported above and each calculated LRI has been compared with those reported in Nist Chemistry WebBook. All analyses were carried out in triplicate.

### 4.8. Broth Microdilution Susceptibility Test

According to the European Committee on Antimicrobial Susceptibility Testing (EUCAST) international guidelines, broth microdilution susceptibility tests were performed in 96 well-plate to evaluate the antifungal activity of CC-EO, CIN, and nano formulations of both (nano-CC-EO and nano-CIN). Briefly, 50 μL of a cell suspension equal to 1 × 10^6^ cfu/mL were added to 50 μL of broth in which a known concentration of CC-EO, CIN or their nano-formulations was previously suspended. Starting from each natural product or nano-formulations, serial dilutions ranging from 0.6% *v*/*v* (6 mL/L) to 0.001% *v*/*v* (0.01 mL/L) were performed. Next, 0.05% *v*/*v* of Tween 80 was utilized as emulsifier, and the plate was incubated for 24 h at 37 °C. Positive controls were included. Minimum inhibitory concentration (MIC) values were visually determined. The MIC was defined as the lowest concentration with total inhibition of growth compared with the growth of positive control. The minimum fungicidal concentration (MFC) was evaluated by seeding and incubating 5 μL of the content of each well on Sabouraud Dextrose agar plates at 37 °C for 24 h. The MFC was defined as the lowest concentration with the death of almost the 99.9% of the initial inoculum. All tests were performed in triplicate.

### 4.9. Checkerboard Test

The checkerboard titration method was used to study the interaction between free or nano-encapsulated CC-EO with fluconazole or micafungin. Specifically, the following concentration ranges were tested: from 0.25% *v*/*v* (2.5 mL/L) to 0.002% *v*/*v* (0.02 mL/L) for CC-EO, from 0.2% *v*/*v* (2 mL/L) and 0.002% *v*/*v* (0.02 mL/L) for nano-encapsulates, from 256 μg/mL and 0.015 μg/mL for fluconazole, and from 4 µg/mL and 0.003 µg/mL for micafungin. Tests were performed using 96-well microplates (Falcon, Corning incorporated, New York, NY, USA), and incubated for 24 h at 37 °C. At the end of the incubation time, the MIC values relative to the single compounds and their synergies were measured to calculate the Inhibiting Fractional Concentrations (FIC) values and the related Indices of Inhibiting Fractional Concentrations (FICI) in accordance with the EUCAST international guidelines (22). The interactions between the various compounds were evaluated with to the FIC or FICI values as follows: synergy (FIC/FICI ≤ 0.5), additive effect (0.5 < FIC/FICI ≤ 1), indifference (1 < FIC/FICI ≤ 2), and antagonism (FIC/FICI ≥ 2).

### 4.10. Biofilm Eradication

To evaluate the ability of CC-EO to disaggregate the biofilm, a suspension of 0.5 Mc Farland of a representative clinical high biofilm producer strain of *C. auris* (strain CA10) was diluted in RPMI broth (LB, Sigma-Aldrich St. Louis, Missouri, USA) in order to have 1 × 10^7^ cfu/mL. The strain was grown overnight in 96-well plates (Thermo Fisher Scientific, MA, USA), at 37 °C for 4 days. After the incubation period, the biofilm was treated for a further 24 h with or without CC-EO. Subsequently, biofilm was washed three times with PBS and cells fixated in acetone for 10 min. Each treatment was eightfold. The following treatments were studied: 256 μg/mL of Fluconazole, 8 μg/mL of Micafungin, and 0.02% *v*/*v* of CC-EO free and nano-encapsulated. The CNT NT was included. Crystal violet staining (Sigma-Aldrich, UK) was used to stain the resultant biofilm for 30 min. Finally, PBS was used to wash the biofilm, ethanol (100 μL/well) was added to completely dissolve the crystal violet, and the absorbance at 560 nm was detected.

### 4.11. Statistical Analysis

Normal distribution data were analyzed using mean and standard deviation parameters. The GraphPad Prism v.8 software (GraphPad Software Inc., San Diego, CA, USA) was used to perform statistical analysis. An ordinary Dunnett’s (*p* < 0.05) multiple comparison test was used to evaluate the effectiveness of nano-CC-EO vs. CNT NT on the biofilm demolition assay.

## 5. Conclusions

CC-EO is an essential oil extracted from a cheaper species of the more valuable species of *C. zeylanicum*. Furthermore, in the extraction phase, the first has a higher yield than the last one and from both EOs with a superimposable chemical profile are extracted. Our data demonstrate that, against *C. auris,* CC-EO has interesting fungicidal activity both when used in free form and encapsulated in PCL particles. In fact, the encapsulation process does not alter the antimicrobial effectiveness of the EO and can contribute to a better delivery and lower toxicity of this EO consisting of cinnamaldehyde as the active compounds. To the best of our knowledge, this study demonstrates for the first time the potential efficacy of CC-EO in the fight against *C. auris* both when used as it is and encapsulated in PLC particles.

## Figures and Tables

**Table 1:** Physicochemical characterization of nano-CC-EO and nano-CIN.

	Z-Average Diameter (nm)	PDI	ζ (mV)	EE%	LC%
nano-CC-EO	203 ± 1	0.12 ± 0.02	−20 ± 2	81 ± 2	54 ± 2
nano-CIN	201 ± 1	0.08 ± 0.01	−11 ± 3	73 ± 6	51 ± 4

**Table 2:** Chemical composition (relative percentage mean values ± standard deviation) of *C. cassia* EO.

N°	COMPONENT ^1^	LRI ^2^	LRI ^3^	CC-EO ^4^
1	benzaldehyde	942	945	0.2 ± 0.01
2	camphene	971	968	0.1 ± 0.02
3	*β*-pinene	990	986	tr
4	p-cymene	1022	1021	tr
5	salicylaldehyde	1030	1026	0.1 ± 0.01
6	1,8-cineole	1032	1026	tr
7	acetophenone	1075	1071	tr
8	phenylethyl alcohol	1105	1102	0.4 ± 0.02
9	endo-borneol	1165	1160	0.1 ± 0.01
10	*α*-terpineol	1190	1186	tr
11	benzene propanol	1210	1205	0.6 ± 0.03
12	*cis*-cinnamaldehyde	1214	1215	0.5 ± 0.02
13	benzaldehyde, 2-methoxy	1244	*	0.1 ± 0.01
14	*trans*-cinnamaldehyde	1280	1275	85.5 ± 1.32
15	eugenol	1335	1331	tr
16	2-methoxyphenylacetone	1340	*	0.4 ± 0.3
17	*α*-copaene	1390	1385	0.5 ± 0.4
18	(*E*)-cinnamyl acetate	1442	1439	tr
19	*α*-curcumene	1490	1485	0.2 ± 0.01
20	*γ*-muurolene	1492	1486	0.2 ± 0.01
21	*β*-bisabolene	1498	1495	0.2 ± 0.01
22	*α*-muurolene	1504	*	0.1 ± 0.01
23	*O*-methoxycinnamaldehyde	1510	1505	8.6 ± 0.15
24	*O*-ethoxy cinnamic acid	1520	*	0.9 ± 0.05
25	*δ*-cadinene	1532	1530	0.6 ± 0.04
26	spathulenol	1611	1601	0.2 ± 0.01
27	*δ*-cadinol	1650	*	0.1 ± 0.01
28	*α*-bisabolol	1677	1674	0.1 ± 0.01
29	benzyl benzoate	1742	1739	0.1 ± 0.02
	SUM			99.8

^1^ the components are reported according to their elution order on apolar column; ^2^ linear retention indices measured on apolar column; ^3^ linear retention indices from literature; * LRI not available; CC-EO ^4^: percentage mean values of pure “*C. cassiae*” EO components; tr: traces (mean value < 0.1%).

**Table 3:** Chemical volatile composition (relative percentage mean values ± standard deviation) of pure and nano-encapsulated *C. cassia* EO.

N°	COMPONENT ^1^	LRI ^2^	LRI ^3^	CC-EO ^4^	Nano-CC-EO ^5^ (%)
1	benzaldehyde	943	945	2.4 ± 0.02	-
2	sabinene	970	972	0.1 ± 0.01	-
3	*p*-cymene	1022	1021	0.1 ± 0.01	0.2 ± 0.01
4	limonene	1025	1026	0.1 ± 0.01	-
5	2-hydroxybenzaldheyde	1026	1027	0.2 ± 0.01	-
6	1,8-cineole	1031	1027	2.5 ± 0.02	11.0 ± 1.05
7	*γ*-terpinene	1058	1054	-	0.1 ± 0.01
8	linalool	1098	1058	tr	0.7 ± 0.06
9	benzenethanol	1105	1102	1.2 ± 0.03	-
10	hydrocinnamaldehyde	1119	1123	-	0.3 ± 0.02
11	borneol	1160	1163	0.5 ± 0.02	0.4 ± 0.03
12	terpinene-4-ol	1180	1174	-	0.1 ± 0.01
13	α-terpineol	1190	1183	0.2 ± 0.01	0.2 ± 0.01
14	*p*-actyltoluene	1195	1190	0.1 ± 0.01	-
15	*cis*-cinnamaldehyde	1211	1215	1.2 ± 0.04	-
16	benzaldehyde, 2-methoxy	1220	*	1.0 ± 0.03	-
17	*p*-anisaldehyde	1233	1229	0.1 ± 0.01	0.5 ± 0.02
18	*trans*-cinnamaldehyde	1280	1275	80.7 ± 2.5	80.1 ± 2.8
19	eugenol	1334	1331	4.3 ± 0.5	2.0 ± 0.04
20	*α*-copaene	1389	1392	2.0 ± 0.02	1.3 ± 0.02
21	*β*-caryophyllene	1426	1440	tr	0.6 ± 0.03
22	*γ*-gurjunene	1483	1479	-	0.1 ± 0.01
23	α-curcumene	1486	1485	0.3 ± 0.02	-
24	α-farnesene	1488	1486	0.1 ± 0.01	-
25	*γ*-muurolene	1490	1487	0.1 ± 0.01	-
26	germacrene D	1495	1489	0.4 ± 0.01	-
27	*β*-bisabolene	1504	1500	0.3 ± 0.02	0.5 ± 0.02
28	*α*-muurolene	1525	*	0.2 ± 0.01	-
29	*O*-methoxycinnamaldehyde	1508	1512	1.0 ± 0.06	0.7 ± 0.01
30	*δ*-cadinene	1535	1530	0.7 ± 0.02	1.2 ± 0.03
31	*α*-bisabolol	11670	1674	0.1 ± 0.01	-
	SUM			99.9	100.0

^1^ the components are reported according to their elution order on apolar column; ^2^ linear retention indices measured on apolar column; ^3^ linear retention indices from literature; * LRI not available; CC-EO ^4^: percentage mean values of pure “*C. cassiae*” EO components; nano-CC-EO ^5^: percentage mean values of encapsulated *“C. cassiae”* EO components; tr: traces (mean value < 0.1%).

**Table 4:** MIC90 and MBC90 values of the three fractions and nano-formulation obtained from CC-EO against ten *C. auris* strains.

Sample	Average (% *v*/*v*) ± St Dev	
	MIC	MFC
CC-EO	0.01 ± 0.01	0.01 ± 0.01
CIN	0.02 ± 0.01	0.02 ± 0.01
Nano-CC-EO	0.02 ± 0.01	0.02 ± 0.01
Nano-CIN	0.01 ± 0.01	0.02 ± 0.00

**Table 5:** Checkerboard titration test between free and nano-incapsulated CC-EO and Fluconazole or Micafungin.

			MIC		Combination		FIC		FICI
CC-EO	AF	SS-AF	CC-EO *	AF ^+^	CC-EO *	AF ^+^	CC-EO	AF	
CC-EO	Fluco	R	0.03 ± 0.01	n.c.	0.03 ± 0.01	n.c.	1 ± 0.00	n.c.	n.c.
CC-EO	Mica	S	0.01 ± 0.01	0.06 ± 0.00	0.01 ± 0.01	0.07 ± 0.08	0.60 ± 0.37	0.60 ± 0.47	1.00 ± 0.70
CC-EO	Mica	R	0.01 ± 0.01	n.c.	0.01 ± 0.01	0.01 ± 0.03	0.50 ± 0.40	n.c.	n.c.
Nano-CC-EO	Fluco	R	0.02 ± 0.01	n.c.	0.01 ± 0.00	19.75 ± 20.50	0.43 ± 0.12	n.c.	n.c.
Nano-CC-EO	Mica	S	0.04 ± 0.08	0.07 ± 0.02	0.02 ± 0.01	0.07 ± 0.03	0.50 ± 0.23	1.00 ± 0.18	1.50 ± 0.39
nano-CC-EO	Mica	R	0.05 ± 0.04	n.c.	0.02 ± 0.01	n.c.	0.38 ± 0.16	n.c.	n.c.

Note: the table shows the average values. CC-EO: *C. cassia* EO; nano-CC-EO: *C. cassia* EO nano-formulated; AF: antifungal drug; SS-AF: strain’s susceptibility against antifungal drug. *: % *v*/*v*, ^+^: μg/mL, n.c.: not countable.
